# Hospital Residents and Hospital Abuse

**Published:** 1907-04-13

**Authors:** 


### HOSPITAL RESIDENTS AND HOSPITAL ABUSE.

Scarcely a number of any of the leading medical papers appears without some reference to the question of hospital abuse. Distant echoes of this controversy are now and then to be heard in the columns of the lay press; but the educated public really takes little interest in a problem which it appears to regard as beyond its province, and of practical interest only to the medical profession. The resident staffs of our London hospitals have at present a tendency to regard this important question from a similarly detached and selfish standpoint. Not being themselves immediately concerned with the evil consequences of this excessive free treatment, they close their eyes to what is going on, and excuse themselves for their inactivity by a protestation that, from the nature of their office, they are powerless to help. It is true that some few of their number do now and then consider the riotous waste of charity that surrounds them. But they receive little encouragement, either from the results of their single-handed and occasional efforts, or from the attitude of the hospital authorities to whom they are responsible. And their colleagues usually shrug their shoulders and regard any action on their part as little better than tilting at windmills.

All this is, of course, utterly wrong. Isolated efforts are certainly useless; but united action by the resident medical officers of all the hospitals of London can, and will, supply almost the only practicable solution of the problem of the abuse of charity. When once this is realised and acted upon, there will be little need for all this discussion and vituperation in the medical press.

In nearly every hospital of importance there is an almoner or inspector—at any rate, an official of some sort—whose duty it is to scrutinise every applicant for out-patient treatment and to inquire into his means and circumstances. In some cases this is efficiently carried out; in others it is a perfunctory business, almost approaching to a farce—a stereotyped question, a lying or evasive answer, and no further inquiries. Where the inspector is conscientious and exacting, the co-operation of the casualty officers and resident staff is all that is needed for the sorting out of the sheep from the goats, and the proper administration of the charity. But in hospitals in which the almoner is slovenly, unobservant, or weak-minded, resolute action on the part of the medical officers forms the only safeguard against excessive and unjustifiable free relief.

If the resident staff of a hospital realise that, in treating out-patients whose incomes enable them to consult a private doctor, they are defrauding the general practitioner and pauperising the patient, their attitude towards this class of person will at least be one of hostility. If they further realise that in actually refusing to treat such patients—except in cases of emergency, or where a card is brought from the medical attendant—they will be supported by their colleagues and by the rest of the profession, they will soon bring these parasites to a proper state of mind, and will eventually force their legitimate views upon the weaker members of the governing body of their hospital.

At the present time it needs some little courage for a house physician, say, to refuse treatment to an able-bodied mechanic, earning £2 or £3 a week, who "isn't satisfied" with his doctor's treatment of his cough or his back-ache. If he has not to face the house committee for thus overstepping the bounds of his duties, he is certain of the mortification of knowing that his would-be patient will be more successful in obtaining treatment and advice at the next hospital to which he chooses to apply.

Generally speaking, the medical officer has better opportunities for gauging the real means of his patients than has the official almoner at the door. The patient's clothing, as he strips for examination, his unguarded answers to astute questions, his speech and his manner, all give indications of the real state of his income. Genuine poverty has a hall-mark which even the freshest of house-surgeons can appreciate.

The worst offenders are usually those who arrive at the hospital doors during the afternoon and at odd times. They are too wary to come during regular casualty hours. They are thus beyond the reach of the official inspector, and their treatment or dismissal lies entirely in the hands of the medical officer on duty. Plausible as their stories often are, there is generally something about them which gives the lie to their statements. Some of them even come long distances from the country without the knowledge of their regular medical attendants, under the mistaken idea that a London hospital is the proper place to apply for a gratuitous and illicit second opinion.

The resident officers of all great hospitals will find that the little extra time and trouble which a critical attitude towards this class of patient entails will be amply repaid. Apart from the knowledge that they are furthering the proper administration of charity, they will have more leisure for the careful and profitable investigation of deserving cases, and they will have the satisfaction of knowing that they are helping those members of their profession whose incomes depend upon the ailments of the lower classes.

A resolute front towards hospital abuse is quite possible. The writer of this article himself adopted it persistently, so far as he could, for many months, and with little addition to his ordinary duties, as a member of the junior staff of two of the larger London hospitals. But he realised that—save as an experiment and as an example—this course of action was of very little use, for lack of support from his own colleagues and from the residents in other hospitals.

The question of in-patients who receive free treatment—operative, medicinal, nursing, and dietetic—and who make absolutely no return to the hospital which does all this for them, is beyond the scope of the present article. But it deserves equally close attention from resident medical officers; for the remedy for this abuse also lies mainly in their hands. This subject will be dealt with in a subsequent issue, and the methods of applying the remedy will be indicated and discussed. In the meantime it is hoped that hospital residents will take the whole subject of the abuse of charity seriously to heart; not only as it affects themselves now and in the future, but also in its widest sociological aspects, as becomes the members of a learned and enlightened profession.

